# Editorial: Immune determinants of COVID-19 protection and disease: A focus on asymptomatic COVID and long COVID

**DOI:** 10.3389/fimmu.2023.1185693

**Published:** 2023-03-28

**Authors:** Maria J. Polyak, Levon Abrahamyan, Mariana G. Bego

**Affiliations:** ^1^ Department of Microbiology, Immunology, and Infectious Diseases, Cumming School of Medicine, Calgary, AB, Canada; ^2^ Faculty of Veterinary Medicine, University of Montreal, Saint-Hyacinthe, QC, Canada; ^3^ BioMedicine Design, Pfizer (United States), New York, NY, United States

**Keywords:** COVID - 19, SARS – CoV – 2, immune activation, long Covid, asymptomatic

SARS-CoV-2 infection manifests as variety of disease presentations, from asymptomatic to mild-moderate COVID-19 symptoms, life-threatening disease, or even persistent debilitating symptoms in some cases (1). Asymptomatic infection occurs in a significant fraction of individuals, and as many as half of all transmission events were reported to occur from pre-symptomatic and asymptomatic individuals (2). One of the most important determinants of disease severity is age as individuals over 65 years have the greatest risk of requiring intensive care, while young children seem to be less severely affected (3, 4). The role of imbalanced immune responses in the overall severity of acute COVID-19 is still not clear. Within this context, we launched our Research Topic “*Immune determinants of COVID-19 protection and disease: a Focus on Asymptomatic COVID and Long COVID*” on May 31^st^ 2021 and invited researchers to contribute towards increasing the understanding of the immunological determinants of COVID-19 disease presentation and severity. We received diverse and insightful manuscript applications, of which Frontiers in Immunology published 15 articles from 232 authors of 12 countries. Despite the diversity of this collective venture, the contributions fall into two main areas of research: serological and cellular responses to SARS-CoV-2 infections.

A first line of research includes contributions examining serological correlates of immune protection. In their perspective commentary, Narasimhan et al. highlighted the importance of following serological responses in asymptomatic individuals, as they could be silent reservoirs to propagate the infection. After evaluating the antibody profiles in 272 plasma samples collected from 59 COVID-19 patients (18 asymptomatic patients, 33 mildly ill patients and 8 severely ill), measuring the IgG against five viral structural proteins, different isotypes of immunoglobulins against the Receptor Binding Domain (RBD) protein, and neutralizing antibodies, Liao et al. concluded that the overall antibody response was lower in asymptomatic infections than in symptomatic infections throughout the disease course. Their data suggests that asymptomatic infection elicit weaker antibody responses, and primarily induce IgG antibody responses rather than IgA or IgM antibody responses. Similarly, Wu et al. studied antibody profiles from 25,091 individuals enrolled in a surveillance program in Wuhan, China, and compared 405 asymptomatic individuals who mounted a detectable antibody response with 459 symptomatic COVID-19 patients. The authors observed that, while IgM responses rapidly declined in both groups, the prevalence and durability of IgG responses and neutralizing capacities correlated positively with symptoms. Furthermore, Castillo-Olivares et al. noted statistically significantly higher levels of SARS-CoV-2-specific neutralizing antibodies in severe COVID-19 patients than people with mild or asymptomatic infections in a cohort of patients and health-care workers from the Royal Papworth Hospital in Cambridge, UK. They also showed a positive correlation between severity, anti-nucleocapsid assays and intracellular virus neutralization. Collectively, these findings shed important lights on the specific character of immune response and highlight the importance of immunization of individuals after asymptomatic infections. In terms of evaluating the post vaccination anti-SARS-CoV-2 serological response, Xu et al. followed 61 volunteers after receiving the inactivated CoronaVac vaccine over 160 days. They observed an intense antibody response for the vaccine with over 95% neutralizing seropositivity rate, reaching a peak two weeks post second dose, however, a decline of this response has been observed a week after.

Several studies examined whether previous exposure to unrelated coronaviruses could modulate SARS-CoV-2 infection. An interesting study from Khan et al. followed two groups of individuals who tested negative for SARS-CoV-2 infection at the start of the study: individuals with a previously confirmed Middle East respiratory syndrome (MERS)-CoV infection and a control MERS-negative group. Within these groups, 24% of the previously MERS-positive (82 individuals) and 31% of the MERS-negative group (260 individuals) eventually contracted SARS-CoV-2 infection. Thus, previous MERS infection did not correlate with higher probability of SARS-CoV-2 infection (symptomatic or not), but the risk of COVID-19-related hospitalization in the MERS-CoV-positive group was significantly higher. Of note, there could be an age-bias in the analysis of this cohort, and, as it was previously established, older adults have been disproportionately affected during the SARS-CoV-2 pandemic, including higher risk of hospitalization (4). Tanunliong, et al. analyzed presence of anti-human coronaviruses (HCoV) antibodies in a Canadian cohort comprised of over 900 samples (half of them predating 2020) expanding from children under 5 years of age to older adults of >80 years of age. They quantified IgG antibody against the Spike proteins of seasonal HCoV, including alpha (HCoV-229E, HCoV-NL63) and beta (HCoV-HKU1, HCoV-OC43) viruses, the 2003 epidemic beta coronavirus, SARS-CoV-1 as well as Spike, Nucleocapsid, and the Receptor Binding Domain (RBD) of SARS-CoV-2. They concluded that most people have an HCoV priming exposure by 10 years of age with stable IgG levels thereafter, and that some of these anti-HCoV antibodies can cross-react with SARS-CoV-2 epitopes. Finally, Sim et al. investigated potential functions for these cross-reactive antibodies found in the blood of pre-pandemic elderly people and hypothesize that they likely could have two opposing functions: protecting against and enhancing viral infection.

The second line of research contributions focused on the cellular immune response to infection as the serological correlates mentioned above are a direct consequence of the immune cells’ activation. Cui et al. report on a critical aspect of this response, reviewing the follicular CD4 T cell (Tfh) subsets, their participation in the humoral immune response, and the important role they play in response to SARS- CoV-2. A review of the reported frequencies of the Tfh subsets in SARS-CoV-2 infected individuals indicate that these cells are expanded in individuals with mild/asymptomatic symptoms, while their numbers are reduced, and germinal centers lost in severe patients. The authors speculate that targeting Tfh cells could serve as therapeutic strategy against SARS-CoV-2 infection.

Several research teams have aimed to understand the immune responses leading to better control of SARS-CoV2 infection using discovery-based methods, comparing individuals spanning the entire spectrum of disease severity. Although SARS-CoV-2 has been demonstrated to be highly transmissible, there are individuals who are resistant to infection despite being exposed to the virus. Castelli et al. explored the possibility that genetic factors led to this resistance by genotyping 83 discordant couples, where one member was COVID-19 symptomatic while the other did not get infected for over six months. Whole-exome sequencing revealed a dominance of several HLA-DRB1 variants in symptomatic individuals while HLA-A alleles encoding 144Q/151R were associated with seronegative women. Interestingly, the highest hits were for the genes MICA and MICB involved in immune modulation of natural killer (NK) cells. The authors speculated that the modified expression of these proteins would likely act to downmodulate NK cell cytotoxic activity and increase susceptibility to SARS-CoV-2 infection. Various immune cell subsets and their abundance have also been correlated to disease severity. Wang et al. performed scRNA sequencing on PBMCs from a cohort of individuals varying from healthy to symptomatic patients with severe disease. The results highlighted the importance of innate immunity in antiviral response as there was an increase in mucosa-associated innate T cells and specific NK cell and classical monocyte subsets in asymptomatic individuals. NKT, Treg and myeloid subsets including monocytes and neutrophils were enhanced in symptomatic patients suggesting they contribute to severe outcome. These results were consistent with previous studies (5), which have shown that the severity of disease caused by SARS-CoV2 infection is correlated with an expansion of myeloid derived suppressor cells (MDSC), particularly the immunosuppressive low-density neutrophil (LDN) subset of these cells. Sieminska et al. were curious if this or other MDSC subsets would result in immunosuppression or long COVID in convalescent individuals who had been infected with SARS-CoV2 but were either asymptomatic or had mild symptoms. They showed that LDN/MDSCs continued to be transiently elevated 35 days after infection, and that the low levels of CD8+ T cells had an exhausted phenotype. The LDN/MDSCs as well as normal density neutrophil subsets expressed PD-L1 and not only affected the production of neutralizing antibodies but also inhibited proliferation of T cells. Together, these results suggest that neutrophil dysfunction is responsible for long-term immunosuppression.

Several research groups have studied the possibility that SARS-CoV2 infection could lead to different outcomes in patients having suppressed or deficient immune systems, e.g., in HIV-1-positive individuals, or those with other types of immunodeficiencies (primary and secondary) (6). This investigation could be relevant in areas with an extremely high prevalence of HIV-1 (e.g., Sub-Saharan Africa) and low level of vaccinations against SARS-CoV-2 – a problem reviewed by Mandala and Liu.

The study of asymptomatic or mild disease has increased our understanding of the immune responses important for protection from SARS-CoV2, since the individuals who are asymptomatic or have mild disease are less likely to be monitored. Soares-Schanoski et al. followed a cohort of United States marine recruits who were initially seronegative, with most seroconverting over time. Although similar dynamics in viral load and generation of specific antibody responses were observed, proteomic analysis revealed a difference in asymptomatic individuals vs those having mild symptoms. For instance, chemokines and cytokines associated with the inflammatory response or immune activation were up-regulated in individuals displaying mild symptoms, while asymptomatic individuals had increased levels of analytes such as IL-17C, MMP-10 and Fibroblast Growth Factors, known to be involved in tissue repair and, in some way, in protection against disease.

Viral infection modulates the intracellular environment to escape host response and create favorable conditions for virus production and spread. Thus, it is plausible that even in the case of asymptomatic infections, SARS-CoV-2 will modify the expression of host genes, albeit differently than during a symptomatic infection. Sfikakis et al. performed a genome-wide transcriptional RNA sequencing of whole blood samples obtained from SARS-CoV-2 seropositive individuals, comparing the differential immune responses relative to symptom presentation. The expression of 15 genes was significantly different, eight of which were associated with interferon related signalling pathways. This led the authors to propose that slight differences in the baseline expression of innate immunity-related genes may be associated with an asymptomatic outcome of SARS-CoV-2 infection.

As seen in this collection, the strength and quality of the host immune response plays important roles in COVID-19 presentation and outcome. March 2023 marked the 3-year anniversary since WHO declared COVID-19 to be a pandemic and since then, collectively and cooperatively worldwide, we have gathered an unprecedented number of critical insights into the roles of the immune system in protection and pathogenesis of COVID-19. Despite how far we have come, the COVID-19 pandemic is not over, it remains a major health concern and there are still many unknowns regarding immune determinants of COVID-19 protection.

## Author contributions

All authors contributed to the outline, compiled/wrote the different sections. All authors reviewed and edited for final revisions. All authors approved the final version for publication.
